# The microbiota affects energy production, nitrogen excretion, and sterol metabolism in mosquito larvae

**DOI:** 10.1128/mbio.01035-26

**Published:** 2026-06-12

**Authors:** Ottavia Romoli, Yanouk Epelboin, Viola Pavoncello, Frédéric Barras, Volker Behrends, Mathilde Gendrin

**Affiliations:** 1 Microbiota of Insect Vectors Group, Institut Pasteur de la Guyane 27058, Cayenne, French Guiana; 2 Viruses and RNA interference Unit, Institut Pasteur, Université Paris Cité, CNRS UMR3569 27058, Paris, France; 3 lnserm, UA17, SPAmaz 638330, Cayenne, French Guiana; 4 SAMe Unit, Department of Microbiology, Institut Pasteur, Université Paris Cité, CNRS UMR6047 27058, Paris, France; 5 School of Medicine and Biosciences, University of West London 7364, London, United Kingdom; 6 Faculty of Medicine, Imperial College London 4615, London, United Kingdom; Case Western Reserve University School of Medicine, Cleveland, Ohio, USA

**Keywords:** mosquito, *Aedes aegypti*, larval development, metabolome, GC-MS, microbiota, transient colonization, nitrogen metabolism, TCA cycle, cholesterol, fatty acids, beta-oxidation

## Abstract

Mosquito larvae rely on a living microbiota for normal development because the microbiota supplies essential nutrients, particularly vitamins. Beyond vitamin provision, transcriptomic data suggest that the microbiota also supports other key nutritional processes. Here, we explored these roles by conducting a metabolomics analysis on *Aedes aegypti* third instar larvae following microbiota depletion. We sampled larvae and dissected guts 12 and 20 h post-decolonization, and analysed methanol-soluble metabolites using untargeted gas chromatography–mass spectrometry. Our findings reveal a pronounced impact of gut microbial presence on several metabolites involved in the tricarboxylic acid cycle and the uricolytic pathway. Germ-free larvae also had a lower quantity of cholesterol in their guts, and their long-chain fatty acid profile was altered in their guts and whole larvae. Sterols, including cholesterol, are essential precursors for the production of the molting hormone 20-hydroxyecdysone. We therefore tested how supplementing exogenous cholesterol affects the development of germ-free larvae. The effects proved to be highly concentration-dependent, ranging from a marginally significant increase in successful development to adulthood at low concentrations, to a pronounced developmental impairment at higher concentrations. Moreover, bacteria deficient in fatty acid beta-oxidation had a significantly lower ability to support larval development. Together, the observed alterations suggest that microbiota-deprived larvae exhibit a downregulation of metabolic processes related to energy production, nitrogen excretion, and sterol metabolism, likely due to the absence of microbiota-derived vitamins essential for these central metabolic functions.

**IMPORTANCE:**

Mosquito larvae depend on gut microbiota for normal growth because microbes supply essential nutrients, particularly B vitamins. To explore microbial roles beyond vitamin provision, we analysed metabolic changes in *Aedes aegypti* larvae after microbiota removal using gas chromatography-mass spectrometry. Germ-free larvae exhibited decreased levels of metabolites associated with the tricarboxylic acid cycle and uricolytic pathway, indicating a general slowdown in metabolic activity and nitrogen waste processing. Additionally, the absence of a microbiota affected cholesterol and fatty acid metabolism. To validate these findings, we found that supplementing germ-free larvae with low levels of cholesterol modestly improved their development. In contrast, larvae colonized with bacteria deficient in fatty acid metabolism exhibited significantly reduced developmental success. Overall, the findings show that removing the microbiota downregulates key metabolic pathways related to energy production, nitrogen excretion, and sterol metabolism, highlighting that bacterial vitamins and fatty acid degradation are vital for mosquito larval development and successful transformation into adults.

## INTRODUCTION

Mosquitoes are holometabolous insects undergoing an aquatic larval development and a terrestrial adult phase. Larval development is the life stage in which the mosquito acquires the totality of energy and elements to form a mature adult. These two distinct developmental stages are characterized by intrinsically different environments and nutritional requirements. During adulthood, mosquitoes fly and feed on plant nectar, and females generally ingest blood because they require this nutritionally rich diet for egg production. During larval development, mosquitoes feed on organic debris and uptake microorganisms that colonize their gut and critically support their development (1, 2). Some microorganisms are transmitted from larvae to adults, while others are taken up by adults from their environment. The composition of this microbiota at both larval and adult stages significantly influences physiology, consequently affecting vector competence to pathogens, fecundity, and lifespan (3–9). Importantly, microbiota composition during larval development has been reported to influence adult physiology without influencing adult microbiota composition. This indicates that host-microbe interactions happening during larval development matter for pathogen transmission (8, 10). Hence, the mosquito microbiota has a strong influence on its host throughout the aquatic and aerial phases of its life cycle.

Focusing on larvae, microbes appear to be required for normal development of the host. In the absence of microorganisms, it is only possible to rear larvae in the dark with a special liver and yeast extract-based diet (11) or with a synthetic medium that includes vitamins, salts, and amino acids (12). In contrast, germ-free larvae kept on conventional day/night cycles, whatever the diet, are blocked in their first instar until microbes are added to the environment (2, 11, 12). Such microbes are not particularly specific; several families of bacteria, as well as yeasts and algae, have been found to support larval development, and differences between microbial strains in developmental success depend on several factors such as virulence and colonization success (13–15). The non-specificity in the taxonomic entities favoring development suggested that microbial support is linked to relatively common constituents, and recent work indeed showed that bacterial support on development notably relies on the provision of B vitamins to their host. On one hand, our transcriptomic analysis on germ-free vs. colonized third instar larvae detected an upregulation of genes of the folate (B9 vitamin) biosynthesis pathway, which is incomplete in mosquitoes, and of two folate transporters (4). We observed that after turning germ-free during the third instar, most larvae did not develop to adulthood, but developmental success increased from 12% to 52% upon folate supplementation. On the other hand, Wang et al*.* developed a diet that supports the development of axenic larvae and showed that an essential dietary compound was light sensitive; they identified riboflavin (B2 vitamin) as an essential dietary requirement for axenic larvae (12). They also found that pyridoxine (B6 vitamin) and, to a lesser extent, thiamine (B1 vitamin) and folate were required for larval development, while pantothenate (B5 vitamin), nicotinic acid (B3 vitamin), and biotin (B7 vitamin) were not. Our recent work indicates a positive impact of biotin when supplemented with folate, but that biotin also has a toxic effect at higher concentrations (16).

Considering the importance of bacterial colonization on mosquito metabolism, we decided to study the metabolome of *Aedes aegypti* larvae colonized or not with bacteria, using a similar experimental strategy as for our previous study. It consists in colonizing germ-free first instar larvae with a bacterial strain auxotroph for meso-diaminopimelic acid and D-alanine, two bacterium-specific amino acids (17). Bacteria are lost by most larvae by 12 h after transfer into an environment devoid of both amino acid supplements. Here, we report our data after gas chromatography-mass spectrometry (GC-MS) analysis on gut and whole larva samples collected during the second half of the third instar, that is, 12 h and 20 h after larval transfer to the bacterium- and supplement-free environment. We found that larvae deprived of their microbiota show an increase in metabolites involved in the citric acid (TCA) cycle, a decrease in cholesterol and metabolites involved in the uricolytic pathway, and a general perturbation of fatty acid metabolism. When supplementing cholesterol to germ-free larvae, we detect a marginally significant increase in the development to adulthood at low concentrations, suggesting an involvement of this metabolite in mosquito larval development. Additionally, we demonstrate that bacteria contribute to larval development by degrading and providing fatty acids to the host.

## RESULTS

### Absence of microbiota in mosquito larvae alters central metabolic pathways, including the TCA cycle and uricolytic pathway

**Fig 1:**
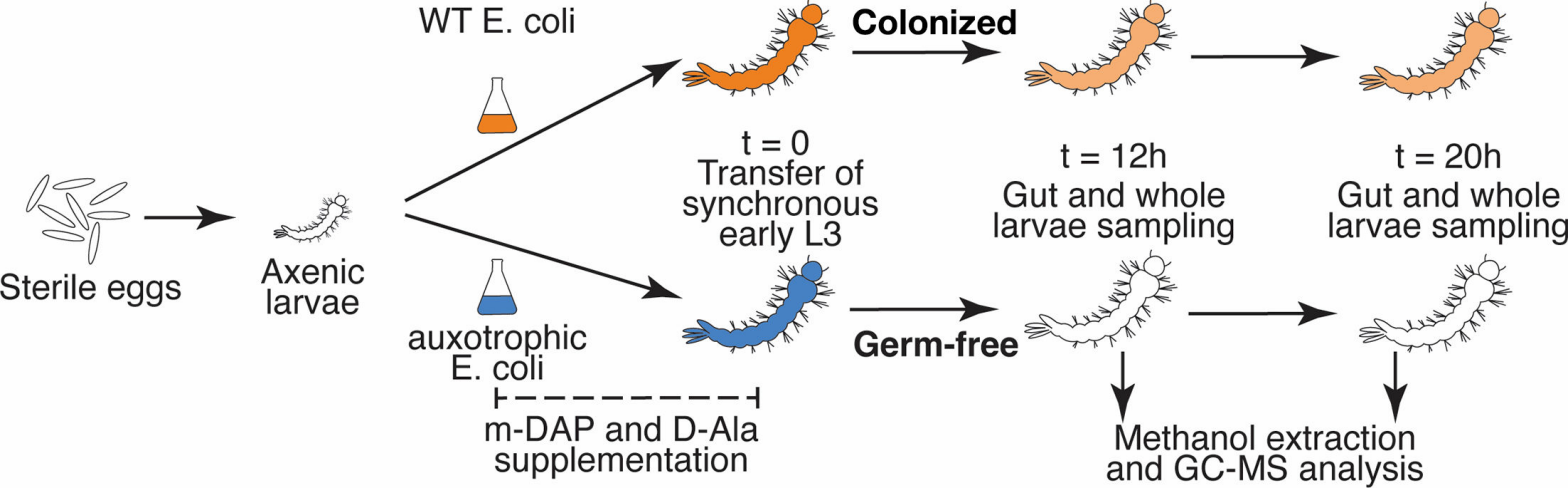
Experimental setup. Axenic *Ae. aegypti* larvae were obtained by egg surface sterilization. To support larval development, larvae were either colonized with wild-type (WT) *Escherichia coli* (orange) or supplemented with an isogenic *E. coli* strain auxotroph (AUX) for the bacterium-specific amino acids meso-diaminopimelic acid (m-DAP) and D-alanine (D-Ala, blue), which were added to the larval water. At the onset of the third instar, both WT- and AUX-colonized larvae were transferred to sterile water containing only sterile fish food (i.e., no bacteria or amino acids). This allowed us to achieve bacterial decolonisation from AUX-carrying larvae and obtain germ-free larvae, while WT-carrying larvae were still colonised by bacteria. Larvae were collected at 12 and 20 h post-transfer for metabolite extraction, from pools of dissected guts and whole larvae. Metabolomic profiles were analysed by gas chromatography–mass spectrometry (GC-MS). The experiment was repeated in four independent replicates, collecting 50–60 individuals per replicate and sample type.

Our previous study on the role of the microbiota in *Ae. aegypti* larvae identified specific transcriptional signatures indicating a strong involvement of bacteria in mosquito metabolism during larval development (4). Thus, we decided to assess the impact of bacterial decolonization on the larval metabolome, using an experimental setup similar to our previous work, that is, focusing on axenic third instar larvae produced via transient colonization with *Escherichia coli* HA416 auxotrophic bacteria. Our control consisted of larvae colonized with the parental WT strain *E. coli* HS. We sampled whole larvae or dissected guts 12 h and 20 h after transfer of early third instar larvae into water that contained fresh sterile food but no bacteria (Fig. 1). As per our previous transcriptomic study, dissected guts were sampled to investigate the processes associated with nutrition, while whole larvae were used to study whole body metabolism. After methanol extraction, we performed a GC-MS analysis of the gut and larval metabolome. This analysis allowed us to specifically quantify 80 metabolites, notably including 17 amino acids, 11 fatty acids, 7 sugars, and other metabolites implicated in diverse pathways (Table S1).

**Fig 2:**
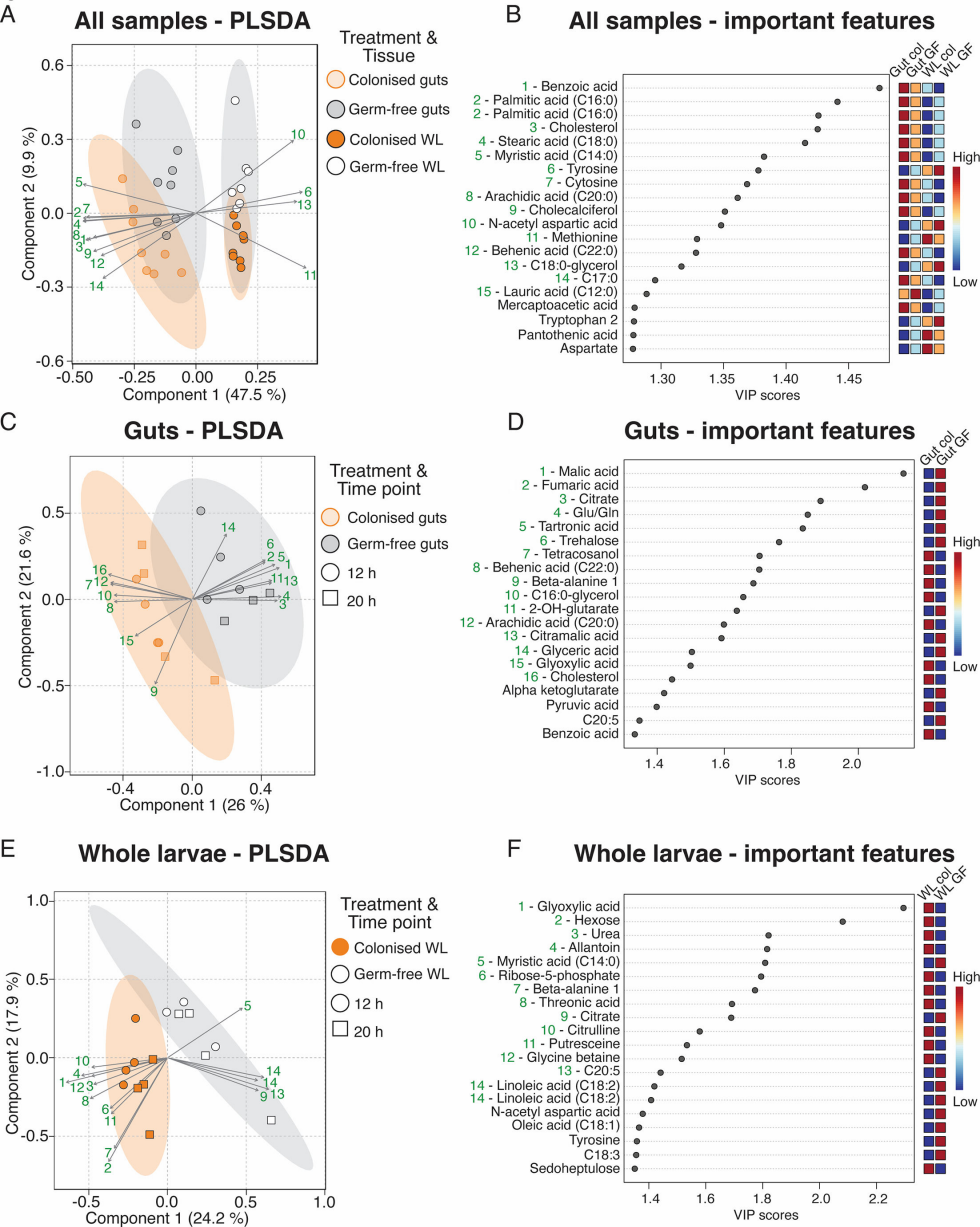
PLS-DA of gut and larval metabolomes. (**A**) PLS-DA, including all samples, showing a clear separation between the gut (transparent symbols) and whole-larva samples (solid symbols). Samples also display partial clustering by colonization status (orange: colonized; white/gray: germ-free). (**B and C**) PLS-DA of gut (**B**) or whole larva (**C**) metabolomes, revealing partial separation according to colonization status (circles: 12 h; squares: 20 h). In all panels, gray and orange ellipses represent the 95% confidence regions for germ-free and colonized samples, respectively. Arrows and green numbers indicate the 15 metabolites contributing most to sample separation; their identities are provided in panels **D–F**. (**D–F**) Key metabolites identified by PLS-DA and their variable importance in projection (VIP) scores for all samples (**D**), gut samples (**E**), and whole-larva samples (**F**). For each metabolite, the mean intensity across sample types is shown in the heatmap on the right.

Partial least squares discriminant analysis (PLS-DA) and principal component analysis (PCA) of all metabolites across all samples revealed a clear separation between gut and larva samples along the first principal component (Fig. 2A; Fig. S1A). Tissue type emerged as the primary explicatory variable driving the differences in metabolome composition, while bacterial colonization had only a marginal impact, and the interaction between colonization and time failed to provide a solid explanation for the variation of metabolite levels (PERMANOVA on tissue PCA, based on Euclidean distance, *F* = 22.7, *P* = 0.001; PERMANOVA on colonization PCA: *F* = 2.0, *P* = 0.085; PERMANOVA on colonization*tissue PCA: *F* = 10.1, *P* = 0.001; PERMANOVA on colonization*time PCA: F = 1.2, *P* = 0.25; Table S2). The important features identified in the PLS-DA were predominantly lipids, fatty acids, sterols, and amino acids, pointing to a different metabolite content of guts compared to whole larva samples (Fig. 2B).

Differences associated with bacterial colonization were significant when focusing on the gut and whole larva samples separately (Fig. 2C ; Fig. S1B, PERMANOVA: *F* = 2.6, *P* = 0.014 on gut PCA; Fig. 2E ; Fig. S1C, PERMANOVA: *F* = 1.8, *P* = 0.039 on whole larva PCA). In gut samples, the metabolites most discriminatory for bacterial colonisation were predominantly TCA cycle intermediates and lipids/fatty acids (Fig. 2D), whereas in whole larva samples, sugars, nitrogen secretion pathway metabolites, and lipids/fatty acids were the key drivers of colonization-related differences (Fig. 2F). Interestingly, time points significantly discriminated whole larva samples (PERMANOVA on PCA: *F* = 2.1, *P* = 0.023), while they did not affect the metabolome structure of gut samples (PERMANOVA on PCA: *F* = 1.0, *P* = 0.39). This contrasts with transcriptomic data, where sampling time points had a strong impact both in guts and larvae (4). Therefore, in the following analyses, we prioritized identifying differences between germ-free and colonized samples over those associated with time points.

**Fig 3:**
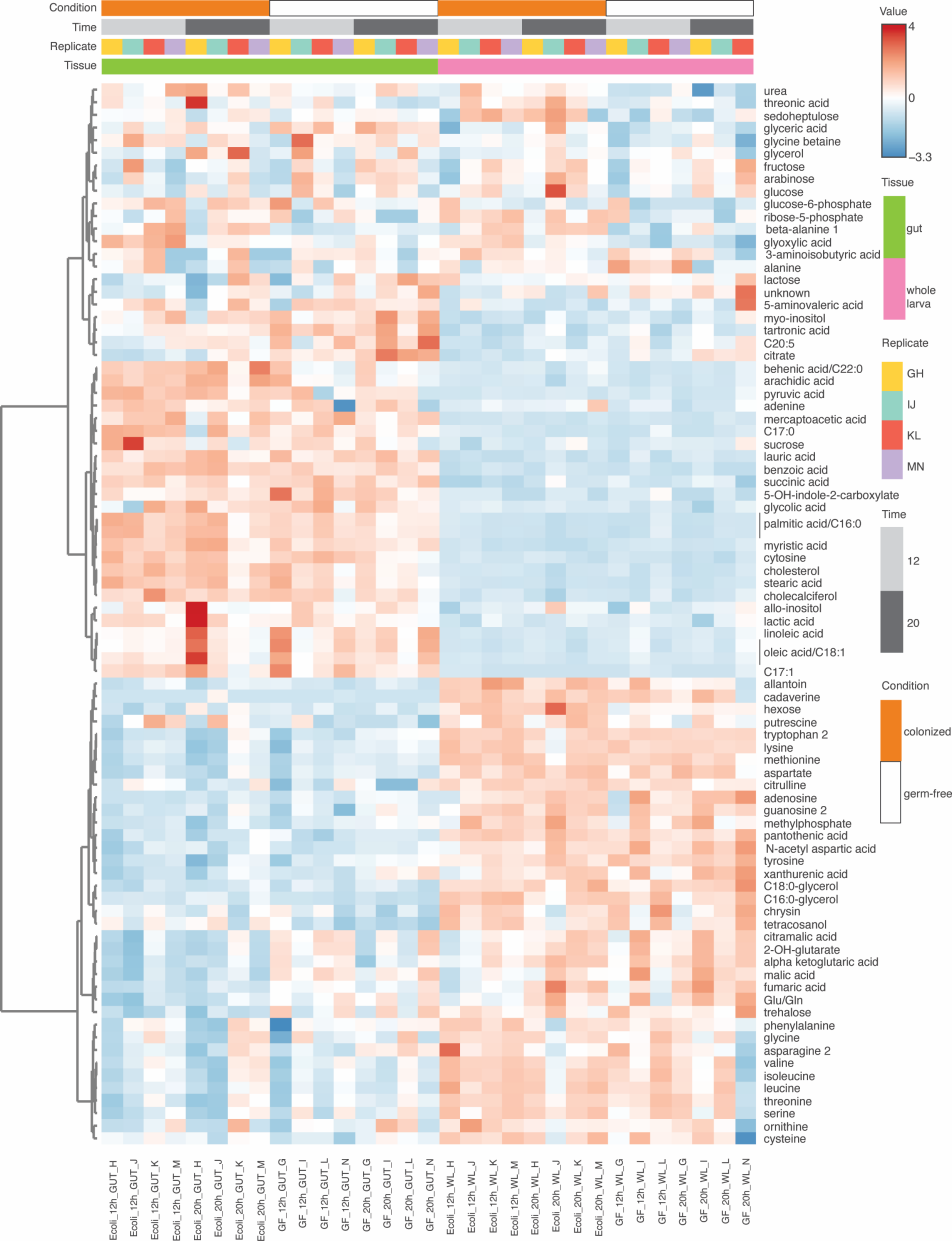
The heatmap of gut and larval metabolomes. The heatmap displays metabolite intensities across samples, organized by colonization status, time point, and tissue type. Hierarchical clustering based on Euclidean distances was performed using Ward’s method. Two primary clusters emerge, reflecting distinct metabolite profiles between gut and whole-larva samples.

Gut and larval samples exhibited distinct metabolite profiles, suggesting the compartmentalization of specific metabolic pathways inside or outside the gut. Specifically, the gut metabolome was enriched in fatty acids (such as lauric acid/C12:0 or palmitic acid/C16:0), while larval samples showed high levels of amino acids (including methionine, tyrosine, and lysine) and monoacylglycerols (such as C16:0-glycerol, Fig. 3).

**Fig 4:**
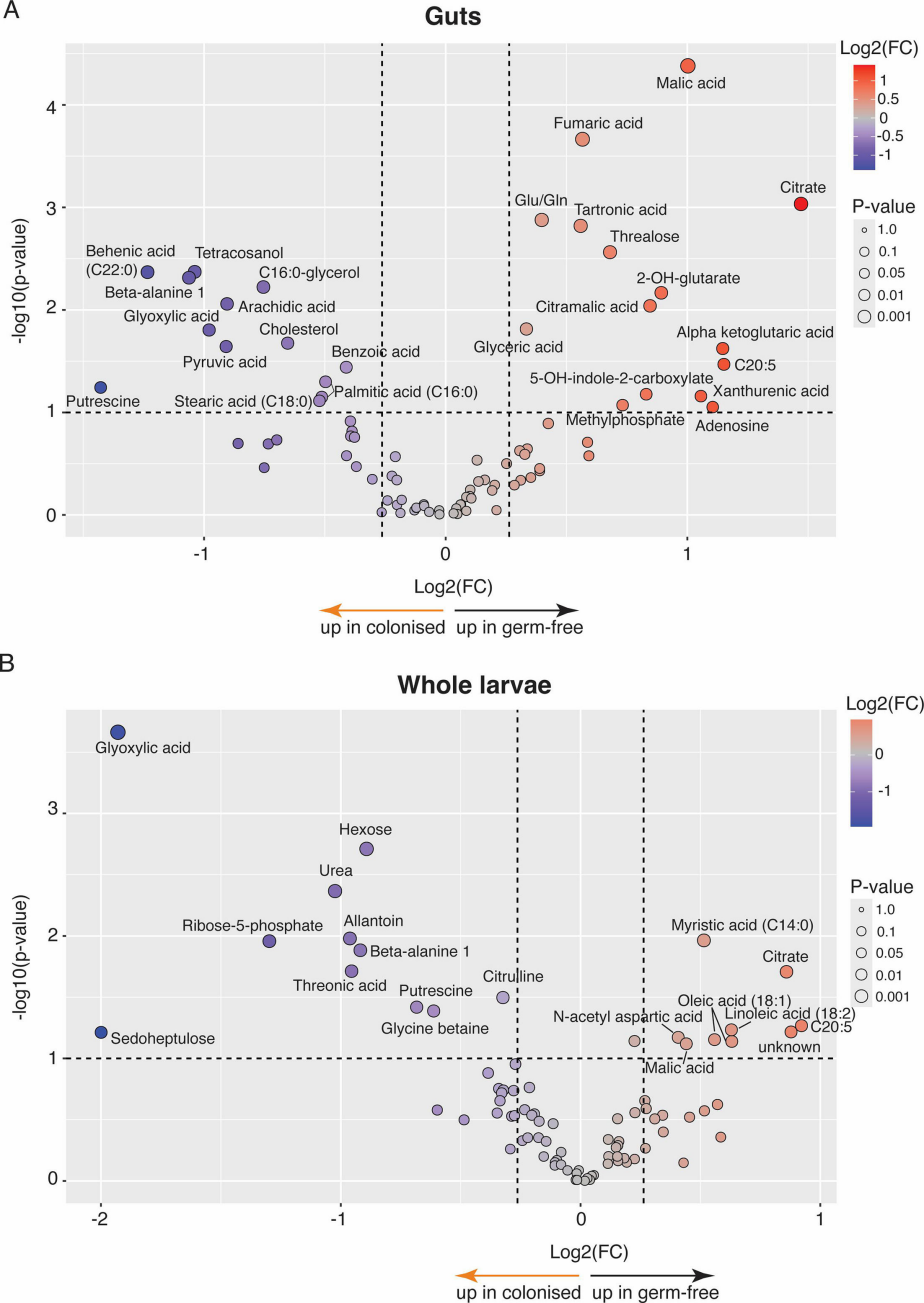
Metabolites significantly affected by bacterial colonization in gut and larval samples. Volcano plots showing differential metabolite abundances between colonized and germ-free conditions in gut (**A**) and whole-larva (**B**) samples. Metabolites enriched in colonized samples are indicated by orange arrows; those enriched in germ-free samples by black arrows. A fold change threshold of 1.2 and a *t*-test *P* value threshold of 0.1 were applied. Both fold changes and *P* values are log transformed for visualization.

Focusing on the impact of bacterial colonization, we observed that 28 and 20 metabolites were significantly affected in guts and whole larvae, respectively, with *t*-test thresholds at *P* = 0.1 and |fold change| = 1.2 (Fig. 4; Fig. S2; Table S2; see Materials and Methods for an explanation of the rationale behind using these thresholds). Levels of several metabolites of the TCA cycle were increased in germ-free conditions—citrate and malate in guts and larvae and alpha-ketoglutarate and fumarate in guts (Fig. 4). Germ-free gut samples also showed a reduced quantity in some lipids and fatty acids (behenic acid [C22:0], arachidic acid [C20:0], palmitic acid [C16:0], and monopalmitoylglycerol [C16:0-glycerol]) or sterols (tetracosanol and cholesterol). On the contrary, other fatty acids were more abundant in germ-free whole larvae, namely, linolenic acid (C18:3), linoleic acid (C18:2), oleic acid (C18:1), and myristic acid (C14:0), while metabolites of the nitrogen homeostasis pathway (urea, glyoxylic acid, and allantoin) and sugars (hexose and ribose-5-phosphate) were depleted (Fig. 4; Fig. S2; Table S2). Germ-free guts and larvae were also deprived of β-alanine, a metabolite involved in the biosynthesis of pantothenate (B5 vitamin).

Finally, differentially abundant metabolites were subjected to a pathway enrichment analysis, which indicated a strong effect of bacterial colonization on (i) TCA cycle (guts: *P* < 0.001, five hits, i.e., five affected metabolites; larvae: *P* = 0.022, two hits), (ii) glyoxylate and dicarboxylate metabolism (guts: *P* < 0.001, seven hits; larvae: *P* = 0.002, three hits), (iii) alanine, aspartate, and glutamate metabolism (gut: *P* < 0.001, six hits; larvae: *P* = 0.041, two hits), (iv) arginine biosynthesis (gut: *P* < 0.001, four hits; larvae: *P* = 0.011, two hits), and (v) synthesis of unsaturated fatty acids (guts and larvae: *P* < 0.001, four hits, Table 1). Additionally, other pathways connected to the TCA cycle were impacted by bacterial colonization in the gut, such as pyruvate metabolism (*P* = 0.003, three hits) and arginine and proline metabolism (*P* < 0.001, four hits).

**TABLE 1:** Pathway enrichment analysis of metabolites affected by bacterial colonization in the gut and larva samples^*a*^

Pathway	Total metabolites	Expected metabolites	Hits	Raw *P*	FDR
Gut (colonized vs. germ-free)					
Glyoxylate and dicarboxylate metabolism	31	0.40	7	4.3E−08	3.4E−06
Alanine, aspartate, and glutamate metabolism	28	0.36	6	6.7E−07	2.7E−05
Citrate cycle (TCA cycle)	20	0.26	5	3.0E−06	7.9E−05
Arginine biosynthesis	14	0.18	4	1.9E−05	3.8E−04
Arginine and proline metabolism	36	0.47	4	9.4E−04	0.013
Biosynthesis of unsaturated fatty acids	36	0.47	4	9.4E−04	0.013
Nitrogen metabolism	6	0.08	2	0.0023	0.027
Pyruvate metabolism	23	0.30	3	0.0028	0.028
Glycine, serine, and threonine metabolism	33	0.43	3	0.0080	0.071
Butanoate metabolism	15	0.20	2	0.015	0.11
Tyrosine metabolism	42	0.55	3	0.016	0.11
Glycerolipid metabolism	16	0.21	2	0.017	0.12
Glutathione metabolism	28	0.36	2	0.050	0.28
Lipoic acid metabolism	28	0.36	2	0.050	0.28
Larvae (colonized vs. germ-free)					
Biosynthesis of unsaturated fatty acids	36	0.42	4	6.1E−04	0.049
Glyoxylate and dicarboxylate metabolism	31	0.36	3	0.0049	0.20
Arginine biosynthesis	14	0.16	2	0.011	0.29
Citrate cycle (TCA cycle)	20	0.23	2	0.022	0.43
Pentose phosphate pathway	23	0.27	2	0.028	0.45
Alanine, aspartate, and glutamate metabolism	28	0.33	2	0.041	0.51
Purine metabolism	70	0.82	3	0.045	0.51

^*a*^ For each pathway, the following elements are indicated: total number of metabolites involved in the pathway, expected number of metabolites found in the pathway, number of metabolites affected by bacterial colonization, raw *P* value, and false discovery rate (FDR). Pathways with an FDR <0.05 are highlighted in gray.

**Fig 5:**
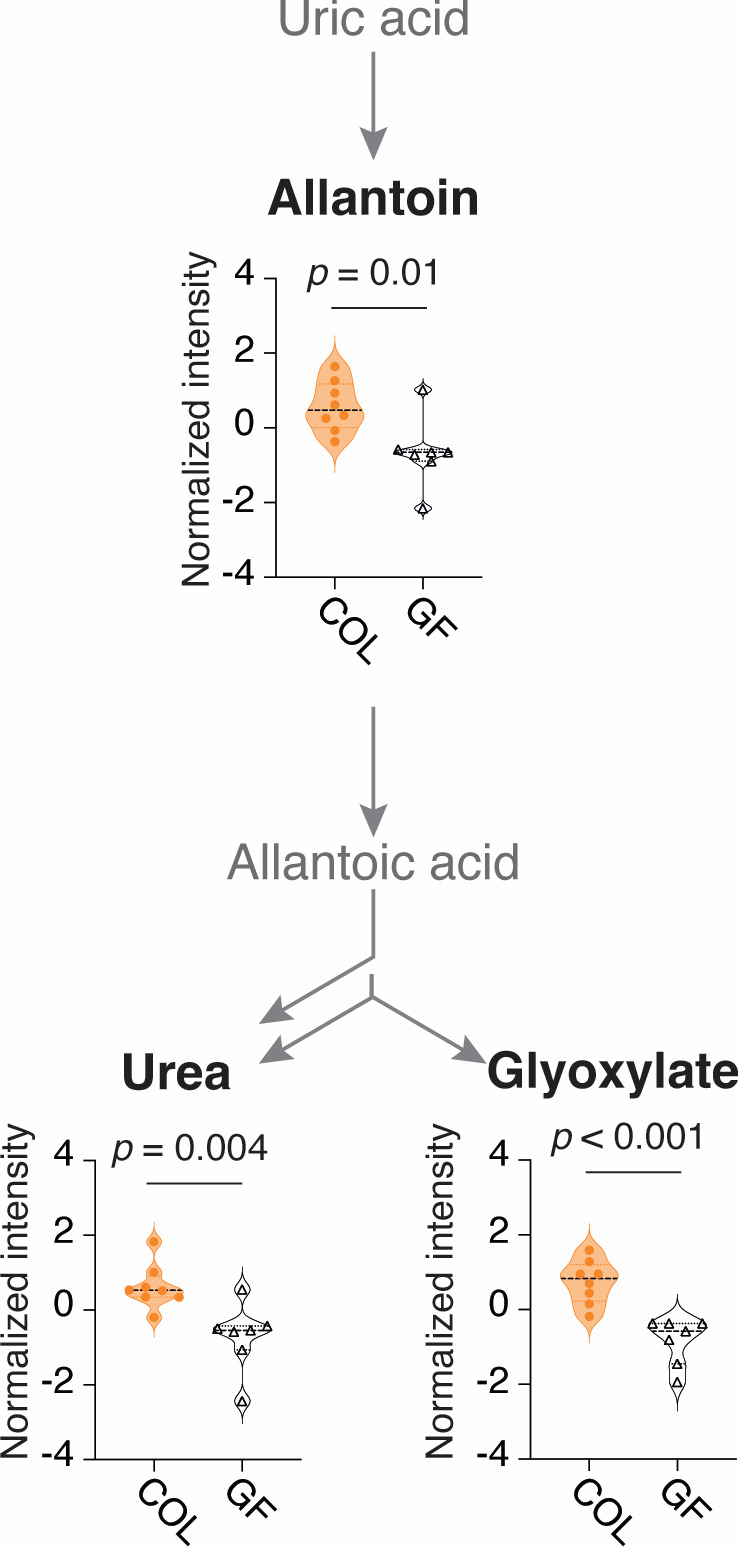
Uricolytic pathway metabolites affected by bacterial colonization in mosquito larvae. Key reactions of the uricolytic pathway are shown: uric acid is oxidized to allantoin, which is subsequently converted to allantoic acid. Allantoic acid is further degraded into two urea molecules and one glyoxylate molecule. Metabolites significantly impacted by bacterial colonization in whole larvae (based on *t*-test) are highlighted in bold; metabolites not detected in our metabolomic data set are shown in gray. For detected metabolites, violin plots display normalized intensities in colonized (COL, orange) and germ-free (GF, white) samples. Median values are indicated by black dashed lines, and quartiles are indicated by orange or black dotted lines.

**Fig 6:**
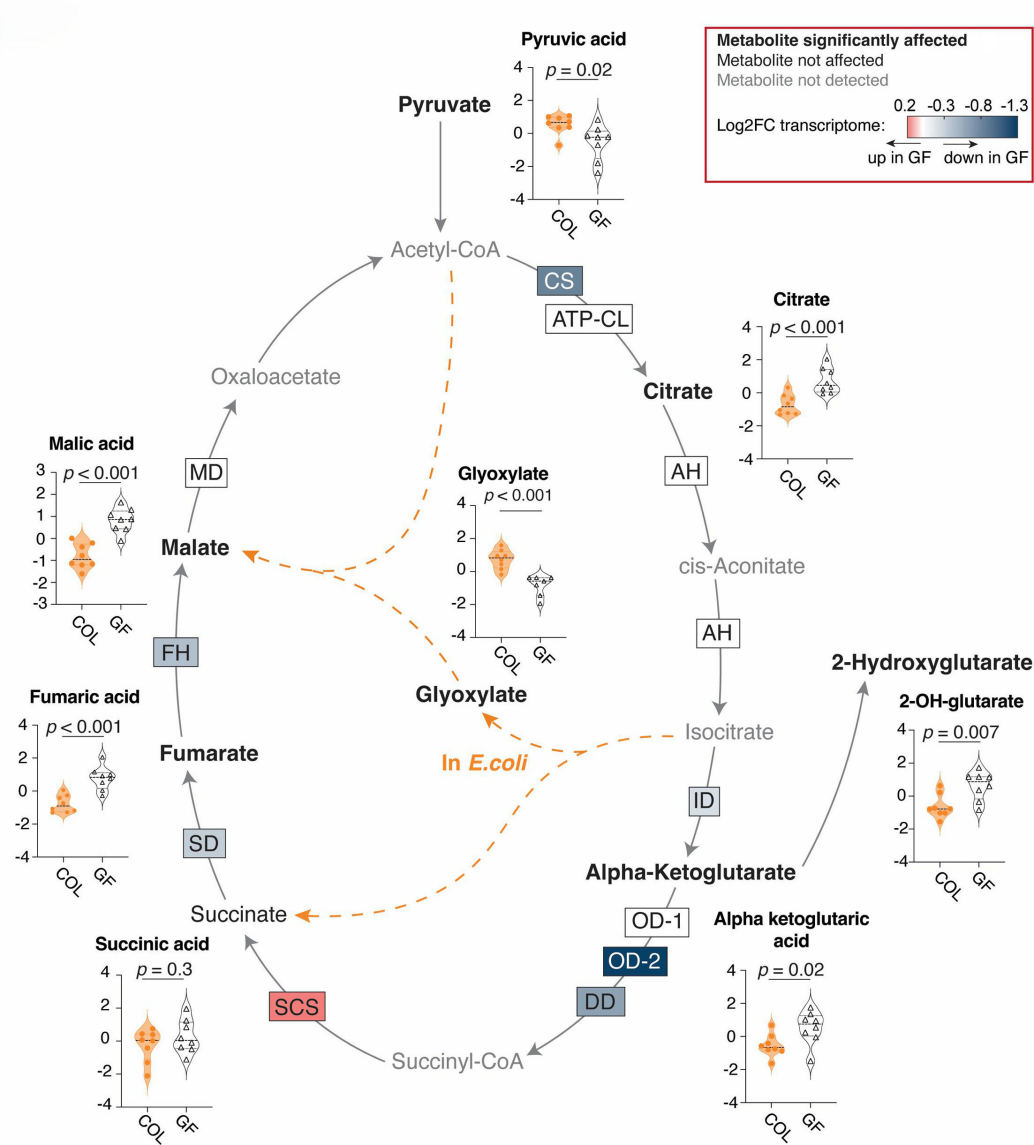
Tricarboxylic acid (TCA) cycle metabolites affected by bacteria colonization in larval guts. Key metabolites and enzymes of the TCA cycle are shown. Metabolites significantly altered by bacterial colonization in larval guts (based on *t*-test) are highlighted in bold, while those not detected in our metabolomic data set are shown in gray. Enzyme boxes backgrounds are color-coded according to GF/WT log_2_ fold change values from our previous transcriptomic analysis (4), averaged across the 12 h and 20 h time points. For detected metabolites, violin plots display normalized intensities in colonized (COL, orange) and germ-free (GF, white) samples. Median values are indicated by black dashed lines, and quartiles are indicatead by orange or black dotted lines. Enzyme names: CS, citrate synthase; ATP-CL, ATP citrate (pro-S)-lyase; AH, aconitate hydratase; ID, isocitrate dehydrogenase; OD-1, 2-oxoglutarate dehydrogenase (E1); OD-2, 2-oxoglutarate dehydrogenase (E2); DD, dihydrolipoyl dehydrogenase; SCS, succinyl-CoA-synthetase; SD, succinate dehydrogenase; FH, fumarate hydratase; MD, malate dehydrogenase.

Overall, these findings highlight a significant influence of the microbiota on the uricolytic pathway in whole larvae (Fig. 5) and on the TCA cycle in guts (Fig. 6). Specifically, several TCA cycle metabolites were more abundant in germ-free guts. Consistent with this, transcriptomic data show the downregulation of multiple genes encoding key TCA cycle enzymes in germ-free guts (Fig. 6) (4).

### Cholesterol affects the development of germ-free larvae in a dose-dependent fashion

**Fig 7:**
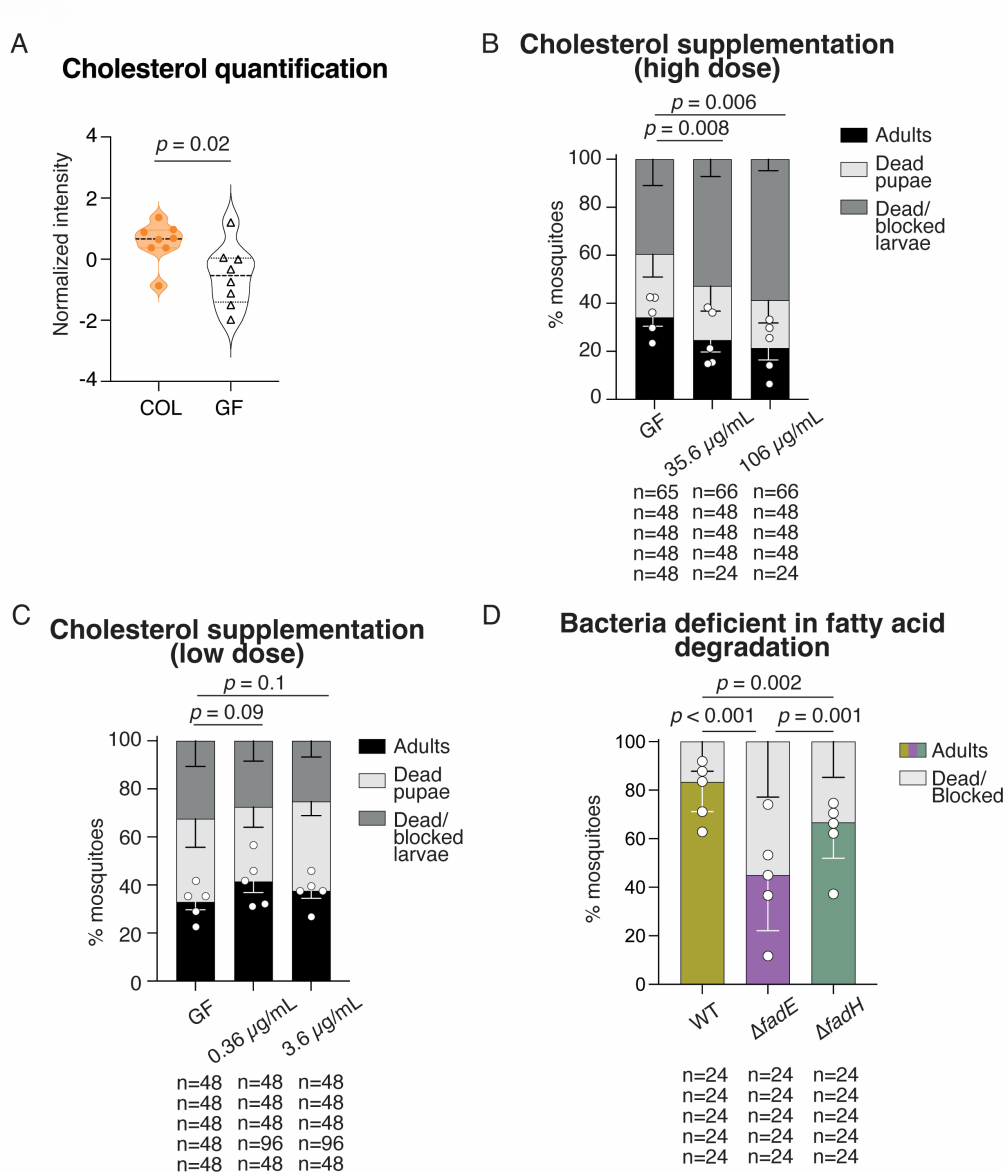
Effect of cholesterol supplementation and bacterial fatty acid degradation on mosquito larva development. (**A**) Normalized cholesterol intensities in colonized (COL, orange) and germ-free (GF, white) larval guts, compared using a *t*-test. Median values are indicated by black dashed lines, and quartiles by orange or black dotted lines. (**B and C**) Developmental outcomes under germ-free conditions with or without cholesterol supplementation. Bar plots show the proportions of adults (black) and developmentally blocked or dead larvae (gray) following supplementation with either high (**B**) or low (**C**) cholesterol doses. (**D**) Developmental outcomes of gnotobiotic larvae colonized by wild-type *E. coli* (WT, khaki) or *E. coli* mutants in *fad*E (purple) and *fad*H (green) genes. Bar plots show the proportions of adults (khaki, purple, or green) and developmentally blocked or dead larvae (gray). All plots show results from five independent replicates. The number of individuals analysed in each replicate is indicated below the plots. Statistical significance on the proportion of adults was assessed using a generalized linear mixed model using the proportion of adults and the cholesterol concentration or bacterial strain as fixed factors and the replicate as a random factor.

Our data set comprises larval and bacterial metabolites whose levels are controlled by multiple pathways, thereby rendering the source of their variation difficult to assess. In contrast, cholesterol, which we found to be depleted in germ-free guts (Fig. 7A), is exclusively derived from the diet (18) and cannot be synthesized by bacteria. Besides its conventional role in membrane homeostasis, cholesterol is crucial for insect development as it is the initial metabolite of the 20-hydroxyecdysone biosynthesis pathway, the insect hormone critical to initiate molting and metamorphosis. Two peaks of ecdysteroids are observed, one during the fourth (and last) larval instar and a stronger one at the beginning of metamorphosis (19). When larvae become germ-free at the start of the third instar, most fail to complete their development, often stalling at the fourth instar or dying (4, 16). We hypothesized that a supplementation of cholesterol may increase the ability of larvae to produce ecdysteroids, hence, to go through the fourth instar development checkpoint. In two sets of experiments, we tested the impact of four cholesterol concentrations on late larval development and metamorphosis of larvae that were decolonized as third instar. In our experimental conditions, larvae were reared individually and provided an equal amount of sterile food. Thus, diet-derived cholesterol can be considered a constant across all conditions, although the absolute cholesterol content of the diet is unknown. First, we added 35.6 and 106 µg/mL, two concentrations that frame those used by Singh and Brown (i.e., 39 µg/mL) to support larval development of germ-free *Ae. aegypti* (20). Cholesterol had a negative impact on larval development at such concentrations (Fig. 7B; generalized linear mixed model [GLMM] on developed individuals: *P* < 0.001; Table S2). Both concentrations significantly impacted the proportion of immatures dead or blocked in their development (GLMM on developed individuals: 35.6 µg/mL, *P* = 0.007; 106 µg/mL, *P* < 0.001; Table S2). A high cholesterol diet is known to have negative impacts on animal health; hence, these concentrations added to the cholesterol already present in the food used to feed larvae probably mimicked the impact of a high cholesterol diet. We therefore lowered the doses of cholesterol supplementation (0.36 µg/mL and 3.6 µg/mL) and tested their impact on larval development. Overall, cholesterol supplementation had a positive, albeit only marginally significant, effect on developmental success (Fig. 7C; GLMM on developed individuals: *P* = 0.074; Table S2). Considering each concentration separately, the effect was again marginally significant at 0.36 µg/mL (GLMM on the proportion of developed adults: 0.36 µg/mL, *P* = 0.09; 3.6 µg/mL, *P* = 0.11). We more specifically noticed that the proportion of mosquitoes dying during metamorphosis was lower in the presence of low cholesterol supplementation, particularly at 3.6 µg/mL (Fig. 7C; GLMM on the proportion of dead pupae, *P* = 0.074; 3.6 µg/mL: *P* = 0.040; 0.36 µg/mL: *P* = 0.27; Table S2).

Overall, our data with lower concentrations suggest that minute supplementation of cholesterol modestly promotes development, potentially by supporting lipid and/or ecdysteroid metabolism, particularly during metamorphosis, while high concentrations mimic the negative impact associated with a high-cholesterol diet.

### Bacterial metabolism and fatty acid degradation are critical for mosquito larval development

The relationship between mosquito larvae and bacteria is complex, as bacteria play a dual role in larval nutrition. On one hand, bacteria can serve directly as a food source: bacterial cells are lysed in the larval gut, and their components and metabolites are taken up and digested to provide energy. This is supported by the observation that axenic larvae can grow and develop when provided with autoclaved *E. coli* agar plugs (i.e., highly concentrated, non-viable bacterial biomass) (11, 12). However, development under these conditions is delayed, suggesting that bacterial metabolic activity also plays a critical role in supporting optimal larval growth.

To investigate this hypothesis, we used our auxotrophic *E. coli* strain HA416, which requires supplementation with meso-diaminopimelic acid (m-DAP) and D-alanine (D-Ala) to grow and, consequently, to sustain larval development (Fig. 1) (4). These molecules are essential components of bacterial peptidoglycan, and in their absence, auxotrophic *E. coli* cannot undergo cell division. When larvae were exposed to a high bacterial dose (~10⁸ CFU/mL) in the presence of m-DAP and D-Ala, nearly all the individuals reached adulthood within a normal developmental timeframe (Fig. S3). In contrast, when the same bacterial dose was provided without these supplements, only ~55% of larvae reached adulthood, with a high proportion dying at the pupal stage (Fig. S3A; GLMM on adult proportion: *P* < 0.001). Additionally, larval development was significantly prolonged (Fig. S3B; linear mixed-effect model on developmental duration: *P* < 0.001). These results indicate that even at high abundance, bacteria are not merely a passive food source; active bacterial proliferation is required to support normal mosquito development. Multiple bacterial metabolic pathways might be essential to mosquito development.

Our metabolomic analyses revealed reduced levels of free fatty acids in germ-free larval guts. This observation may reflect the absence of bacterially derived fatty acids, reduced endogenous fatty acid production from dietary substrates, and/or increased mobilization of host lipid reserves. Reduced production could indicate that in the absence of microbiota, larvae have limited access to fatty acid precursors (e.g., short-chain fatty acids) or exhibit impaired hydrolysis of complex lipids (e.g., triglycerides or cholesteryl esters). Increased consumption of lipid reserves would suggest that germ-free larvae rely more heavily on internal stores to generate essential metabolites such as acetyl-CoA, ATP, or short-chain fatty acids, metabolites that would otherwise be supplied, at least in part, by bacterial metabolism.

To assess the importance of fatty acid degradation by bacteria, we examined *E. coli* mutants deficient in this pathway: Δ*fadE* and Δ*fadH*. FadE is an acyl-CoA dehydrogenase catalysing the first step of β-oxidation, while FadH provides the β-oxidation entry point for the degradation of unsaturated fatty acids (21, 22). Previous work showed that a Δ*fadE* mutant fails to colonize larvae at low inoculum (~10⁶ CFU/mL) but can support development at higher doses (15). In our experiments, both Δ*fadE* and Δ*fadH* mutants, when provided at low doses (~10³ CFU/mL), were recoverable from larval water (Fig. S4A) and fourth instar larvae (Fig. S4B) and exhibited similar proliferation dynamics as their WT control. This indicates that neither mutant displayed a colonization defect under our conditions and that both mutants were actively proliferating in the larval water. Despite this, both mutants significantly impaired larval development at low doses, reducing the proportion of individuals reaching adulthood (Fig. 7D). Notably, the Δ*fadE* mutant had a stronger effect than the Δ*fadH* mutant (GLMM on development success: WT vs Δ*fadE*, *P* < 0.001, ~45% ± 10% of developed mosquitoes; WT vs. Δ*fadH*, *P* = 0.002, ~67% ± 6% of developed mosquitoes; Δ*fadE* vs. Δ*fadH*, *P* = 0.001), consistent with the broader role of FadE in β-oxidation compared to the more specialized function of FadH in unsaturated fatty acid metabolism. This suggests that bacteria have an important role in degrading both saturated and unsaturated fatty acids. At high bacterial doses (~10⁸ CFU/mL), no differences in larval developmental success were observed between strains (Fig. S4C), suggesting that the defects associated with these mutants are not due to reduced bacterial fitness but rather to metabolic limitations. At such high concentrations, the abundance of bacterial metabolites likely compensates for pathway deficiencies. Together, these results demonstrate that bacterial fatty acid degradation contributes significantly to mosquito larval development, highlighting the importance of microbial metabolic activity beyond simple nutritional provisioning.

## DISCUSSION

In this study, we investigated the metabolic consequences of bacterial colonization in third-instar mosquito larvae. Our data reveal microbiota-dependent regulation of several metabolites involved in the TCA cycle and urea synthesis. Additionally, the presence of gut microbes significantly affected the abundance of several lipids, fatty acids, and cholesterol. We further demonstrate that dietary cholesterol supplementation and bacterial fatty acid degradation influence larval development.

This metabolomic study complements our previous transcriptomic analysis of germ-free larvae (4). In both studies, we employed an auxotrophic *E. coli* strain to achieve mosquito decolonization and used the parental wild-type *E. coli* strain as the sole larval colonizer for controls. This choice was motivated by the availability of the auxotrophic strain, which had previously been used to transiently colonize mice and render them conditionally germ-free. We acknowledge that *E. coli* is not an ecologically relevant member of the mosquito microbiota, as it is not typically found among its natural colonizers. Nevertheless, its ability to rescue germ-free larval development as fast as non-sterile larvae suggests that it provides sufficient metabolic support for mosquito growth.

The nutritional requirements of mosquito larvae have been a subject of research interest for over a century (20, 23, 24). Although larvae acquire most of their nutrients from the diet (detritus in natural settings and fish food in laboratory rearing), the microbiota is required to supply essential metabolites that are absent from the diet or to render certain nutrients bioavailable for absorption. Indeed, mosquito larvae cannot develop under axenic conditions when provided exclusively with their standard sterile diet. Over the past century, experiments under sterile conditions using more or less chemically defined diets have sought to identify these essential nutrients and determine their optimal concentrations (1, 12, 23, 24). Although such studies have identified key nutritional requirements, larvae reared in artificial media consistently develop more slowly than those harboring a microbiota (11, 12). These differential developmental rates suggest that some microbiota-derived metabolites or microbiota-activated metabolic pathways remain unidentified or that certain metabolites must be delivered by a living microbiota. A progressive provision of metabolites solves the issues of metabolite decay, for instance, because of light sensitivity, and of their toxicity at high concentrations (12, 16).

We employed GC-MS to characterize the larval metabolome in the presence or absence of *E. coli*, identifying 80 metabolites across our samples. This analytical platform has inherent limitations: only low-molecular-weight volatile compounds are detected, and thermally labile metabolites that degrade at elevated temperatures cannot be analyzed directly. We therefore cannot exclude that biologically relevant metabolites, such as vitamins, may have gone undetected. Nevertheless, we were able to identify four principal metabolic shifts in mosquito larvae deprived of *E. coli*.

In germ-free guts, we detected reduced levels of (i) most TCA cycle metabolites and (ii) fatty acids, sterols, and lipids, whereas in germ-free whole larvae, we observed an increase in (iii) nitrogen waste metabolites and (iv) fatty acids. The interpretation of these results is complicated by the fact that some metabolites may originate from both mosquito and bacterial metabolism and that many of these pathways are deeply interconnected. Moreover, lower levels of a given metabolite or of an entire pathway may indicate either reduced acquisition or availability of that metabolite, or its rapid consumption, further confounding biological interpretation. Additionally, bacteria play a dual role in mosquito nutrition: on one hand, they actively produce nutrients and metabolites essential for larval development; on the other, they serve as a food source, with their fatty acids, sterols, amino acids, and proteins being directly assimilated by larvae. Consistent with this, mosquitoes can develop to some extent when provided with autoclaved agarose plugs containing *E. coli* and liver extract (if protected from light to prevent vitamin degradation) or when provided with proliferation-deficient *E. coli* (Fig. S3), indicating that even dead or stalling bacterial cells in the gut serve nutritional purposes (11, 12).

Empirical observations show that third-instar larvae subjected to bacterial decolonization exhibit a markedly reduced capacity to molt into pupae and reach adulthood (4). This indicates that larvae deprived of their microbiota at this stage lack essential nutrients required for optimal development and can therefore be considered nutritionally deficient. Such a deficiency might result in a general slowdown of larval metabolism or a higher consumption of endogenous reserves. Both these hypotheses may explain the accumulation of TCA cycle intermediates observed in germ-free larvae (Fig. 6). On one side, germ-free larvae assimilate fewer dietary lipids, leading to reduced β-oxidation of fatty acids and consequently lower production of acetyl-CoA. This diminished flux through the TCA cycle could contribute to the accumulation of its intermediates. In addition, deficiencies in microbiota-derived B vitamins may further contribute to reduced TCA cycle activity. For instance, riboflavin is required for the synthesis of flavin adenine dinucleotide (FAD) and flavin mononucleotide (FMN), essential cofactors for multiple enzymes involved in the TCA cycle and oxidative phosphorylation. A shortage of these cofactors would be expected to further limit metabolic flux. Alternatively, the reduced lipid levels observed in germ-free larval guts may reflect a compensatory response to impaired nutrient acquisition. In this scenario, larvae mobilize endogenous lipid reserves, increasing acetyl-CoA production to feed into the TCA cycle, which could also lead to intermediate accumulation. However, the observed downregulation of genes encoding TCA cycle enzymes (Fig. 6) (4), including the rate-limiting step of isocitrate dehydrogenase, supports the interpretation that germ-free larvae primarily experience an overall reduction in metabolic activity and energy production, resulting in decreased TCA cycle turnover and the accumulation of intermediates. The reduction of TCA cycle metabolites we observed in germ-free larvae is in line with our previous metabolomic study of adult *Anopheles* mosquito guts, reared under conventional conditions, and treated or not with antibiotics (25). Thus, bacterial colonization appears to influence central carbon metabolism across developmental stages, with an overall slowdown of the TCA cycle and thus a potential decrease in ATP production in cells.

Considering nitrogen excretion, we detected urea rather than uric acid as the prominent ammonia waste product in larvae, which aligns with previous reports of low uric acid levels in third-instar larvae compared to adults (26). In *Ae. aegypti*, urea can be produced via the hydrolysis of arginine into urea and ornithine or via an amphibian-like uricolytic pathway that successively degrades uric acid into allantoin, allantoic acid, and finally urea and glyoxylic acid (Fig. 5) (27). Here, we identified urea, allantoin, and glyoxylate in larvae, suggesting the uricolytic pathway as the primary route for larval urea production. Lower levels of these nitrogen-waste intermediates in germ-free whole larvae could indicate either accelerated excretion of excess nitrogen, driven by increased protein catabolism to compensate for reduced nutrient intake, or, conversely, a general metabolic slowdown that generates less nitrogenous waste. The latter interpretation is consistent with the observation that larval growth is retarded following bacterial decolonization, concurrent with downregulation of *hexamerin* genes (4). Hexamerins are haemolymph proteins involved in the transport and storage of amino acids in preparation for metamorphosis; their abundance might reflect the extent of amino acid uptake and storage and, by extension, ammonia detoxification via the urea cycle.

Higher levels of certain fatty acids in whole germ-free larvae (e.g., myristic acid, C20:5, linoleic acid, and oleic acid) suggest that in the absence of a microbiota, larvae are arrested in their development and are unable to mobilize their lipid reserves. Consistent with our findings, axenic *Drosophila* accumulate higher fatty acids and triacylglycerol reserves compared with conventionally colonized flies (28, 29), possibly owing to a deficiency in microbiota-derived pantothenate (vitamin B5) (30) and riboflavin (vitamin B2) (29). Pantothenate and its precursor β-alanine are required for coenzyme A (CoA) biosynthesis. CoA is a central cofactor at the intersection of numerous catabolic and anabolic pathways, notably in fatty acid synthesis and β-oxidation and in the TCA cycle. Although pantothenate was not detected in our analysis, we observed reduced levels of β-alanine in germ-free larvae. Riboflavin has already been identified as a key vitamin for mosquito development (12). Its role as precursor of FAD and FMN highlights its importance in all processes requiring FAD-dependent enzymes, such as oxidative phosphorylation and fatty acid β-oxidation. This suggests that, as in *Drosophila*, axenic mosquitoes exhibit altered fatty acid levels, likely because of impaired pantothenate and riboflavin provisioning by the microbiota (29, 30).

The reduced levels of fatty acids in germ-free guts observed in our metabolomic analysis contrast with previous studies reporting lipid droplet accumulation in germ-free larvae and decreased lipid content in the fat body (4, 9, 31). This apparent discrepancy may arise because lipid droplets were visualized using nonspecific lipid staining, which primarily reflects stored neutral lipids rather than free fatty acids—the species quantified in our metabolomic approach. A comprehensive understanding of lipid and fatty acid metabolism in germ-free larvae will require targeted lipidomic analyses of dissected guts and fat bodies.

We chose to further investigate some of these observations and hypotheses through two follow-up experiments. First, we colonized germ-free first-instar larvae with two bacterial mutants deficient in fatty acid β-oxidation. The Δ*fadE* mutant is defective in the first step of β-oxidation, whereas the Δ*fadH* mutant is impaired in the degradation of unsaturated fatty acids. As a result, both mutants are unable to efficiently utilize exogenous fatty acids for growth and may accumulate metabolic intermediates. When provided to larvae at low doses, these mutants show a reduced capacity to support larval development compared with the wild-type strain. Although the diet used in our experiments is chemically undefined, it contains fatty acids (32). Wild-type bacteria likely degrade these dietary fatty acids and supply larvae with β-oxidation products, notably acetyl-CoA, ATP, and short-chain fatty acids, whereas the Δ*fadE* and Δ*fadH* mutants provide lower levels of such metabolites, leading to impaired larval development. In light of these findings, the lower fatty acid levels detected in germ-free larval guts may reflect increased β-oxidation of larval endogenous lipid reserves to compensate for the reduced supply of exogenous β-oxidation products by the microbiota.

The second follow-up experiment involved exogenous cholesterol supplementation after bacterial decolonization. Cholesterol cannot be synthesized by mosquitoes or bacteria, and it is, in principle, absent in bacterial cells. We found that its levels were lower in germ-free guts. Previous studies have demonstrated that cholesterol must be included in chemically defined diets to support germ-free larval development and that the amount of cholesterol present in autoclaved *E. coli* is insufficient to sustain larval growth (12). This indicates that cholesterol is essential for larval development and that although bacteria cannot synthesize it, they may facilitate its bioavailability to larvae. Cholesterol notably serves as a precursor for 20-hydroxyecdysone biosynthesis. Importantly, *sterol carrier protein-2 (AAEL026044)*, which encodes the major cholesterol transporter, is downregulated in germ-free larvae, while the first downstream enzyme in the 20-hydroxyecdysone synthesis pathway is not (4). This suggests that in the absence of a microbiota, larvae sense reduced cholesterol levels. Studies in mammals suggest that the microbiota is involved in host cholesterol metabolism through bile salt hydrolysis or cholesterol conversion (33, 34). Although we did not elucidate the exact mechanism behind our observations, similar bacterial bioconversion or emulsification might render cholesterol more absorbable to mosquito larvae.

Interestingly, a link between ecdysone and fatty acid degradation has been observed in adult female mosquitoes, where silencing the ecdysone receptor reduces fatty acid degradation (35). A causal link may thus exist between our observations, where bacteria would stimulate cholesterol harvest and ecdysone metabolism, which would in turn promote fatty acid degradation; further work is however required to test this hypothesis. We only observed a minor, marginally significant increase in development to adulthood upon cholesterol supplementation to germ-free larvae. Like all holometabolous insects, mosquito larvae must reach a critical weight before initiating metamorphosis (36). Therefore, although cholesterol supplementation may have provided sufficient metabolites for ecdysone synthesis, germ-free larvae potentially lacked other essential nutrients required to attain the critical weight necessary for the commitment for metamorphosis.

In conclusion, while previous data on the role of bacteria in mosquito development primarily focused on vitamin biosynthesis, the use of metabolomics highlighted other aspects of such support. We identified the TCA cycle and urea synthesis as primary pathways affected in mosquito larvae by the absence of a microbiota and detected additional perturbations to lipid metabolism, specifically in cholesterol and fatty acid levels and distribution. The observed low abundance of cholesterol in germ-free guts and a marginally significant increase in development to adulthood upon supplementation, together with its known role in 20-hydroxyecdysone production, point to a role of the microbiota in promoting metamorphosis via cholesterol metabolism. Finally, bacteria-mediated fatty acid degradation appears important for the development of immature mosquitoes. Taken together, these data indicate that mosquito larvae deprived of their microbiota experience a broad metabolic slowdown, likely driven by reduced nutrient uptake and disruption of key metabolic pathways due to the absence of essential metabolites, such as B vitamins.

## MATERIALS AND METHODS

### Bacterial strains and mosquito lines

*E. coli* HS (wild type [WT]) was grown in lysogeny broth (LB). *E. coli* HA416 (auxotroph [AUX] (17) was grown in LB supplemented with 50 μg/mL kanamycin, 50 μg/mL meso-diaminopimelic acid (m-DAP), and 200 μg/mL D-alanine (D-Ala). FBE640 and FBE765 strains were obtained by transducing the ∆*fadE::kanR* and ∆*fadH::kanR* mutations from the KEIO bank into the MG1655 strain (21, 22). Cultures were inoculated from single fresh colonies and incubated at 30°C, shaking at 200 rpm for 16 h prior to centrifugation to set up gnotobiology experiments. They were spun down, and bacterial pellets were diluted five times (high dose) or 500 times (low dose) in sterile milliQ water.

The *Ae. aegypti* New Orleans colony was maintained under standard insectary conditions at 28°C–30°C on a 12:12 h light/dark cycle. Gnotobiotic mosquitoes were maintained in a climatic chamber at 80% relative humidity on a 12:12 h light/dark 30°C/25°C cycle.

### Gnotobiology procedures and sample collection

Gnotobiotic mosquitoes were generated following previously described experimental procedures (4, 37). Briefly, eggs were surface-sterilized using successive 5 min washes in 70% ethanol, 1% bleach, and 70% ethanol and rinsed three times in water and kept in sterile milliQ water overnight. The next day, sterile larvae were individualized in 24-well plates and provided with autoclaved food (Tetramin baby) and a suspension of ~10^8^ CFU/mL HS or HA416 bacteria to produce control and germ-free larvae, respectively. Two days later, larvae were inspected to detect any third instar larvae (L3). They were excluded from the experiment as their age as L3 was undetermined. Then, L3 molted in a time window of 5 h were washed in sterile milliQ water and transferred to 24-well plates in sterile water with fresh food. The latter point is slightly different compared to our transcriptomic study (4), where colonized larvae were not transferred. Although such a transfer temporarily disturbs colonisation in control larvae (4), we thought that it was particularly important for metabolomic analysis that the dietary conditions were exactly the same between control and germ-free conditions. Several sterility checkpoints were used during each experiment. Specifically, water from sterilized eggs and water from the transferred larvae were serially diluted and plated in LB plates. The absence of colonies (for the HA416 auxotrophic *E. coli* strain) or the presence of a single type of colonies (for the HS wild-type *E. coli* strain) were essential conditions to continue further with the experiment or larva collection. Whole larvae or dissected guts were sampled 12 h and 20 h after transfer, corresponding to an early timing after HA416-colonized larvae became sterile (95% at 12 h) (4) and a late timing shortly before L3 would molt to the fourth instar. Bacterial contamination during gut dissection was considered unlikely to significantly alter the metabolome; therefore, dissections were not performed under strict sterile conditions. Nevertheless, basic precautions were observed: larvae were maintained in sealed 50 mL tubes and dissected promptly under a stereomicroscope using glass slides and forceps pre-cleaned with 70% ethanol. Dissected guts were kept around −40°C in 700 µL of 80% methanol during dissection and transferred in a −80°C freezer for a few hours, until sampling was finished, and methanol quenching was performed on the same day. A total of seven independent replicates were performed. However, the first replicate did not include sterility checks, while replicates two and three were contaminated. While all samples were processed and acquired, these first three replicates were excluded from the analysis.

### Methanol quenching

Samples were homogenized at 9,000 rpm for 2 × 60 s in a Precellys Evolution homogenizer (Bertin Technologies), and cellular debris was spun down at 14,000 rpm for 15 min at 4°C. After sampling the supernatant, the pellet was mixed with 700 µL of pre-cooled 80% methanol, homogenized, and centrifuged again. Both supernatants of a sample were pooled and dried in a vacuum drier (Eppendorf) at room temperature until complete solvent evaporation. They were kept at −80°C until lyophilization and shipping with ice packs.

### Dual-phase extraction and sample preparation

Samples were resuspended in 300 μL of CHCl_3_/MeOH (2:1), vortexed for 30 s, and supplemented with 300 μL of water. They were spun at 13,000 rpm for 10 min at room temperature, and the top aqueous layer was then transferred into an inactivated glass vial and dried in a vacuum drier. Samples were stored at −80°C. The lower organic layers were not used in this experiment. For GC-MS, the samples were derivatized by a two-step methoximation-silylation derivatization procedure. The dried samples were first methoximated using 20 μL of 20 mg/mL methoxyamine hydrochloride in anhydrous pyridine at 37°C for 90 min. They were then silylated with 80 μL of N-methyl-N-(trimethylsilyl)trifluoroacetamide (MSTFA) at 37°C for 30 min.

### GC-MS data acquisition

GC-MS analysis was performed on an Agilent 7890 Gas Chromatograph (Agilent, Santa Clara, CA, USA) equipped with a 30 m DB-5 ms capillary column with a 10 m DuraGuard column coupled to an Agilent 5975 MSD system (Agilent, Santa Clara, CA, USA), which operates under electron impact ionization. Samples were injected with an Agilent 7693 AutoSampler injector (Agilent, Santa Clara, CA, USA) into deactivated splitless liners, following a temperature gradient detailed in a previous study (38), using helium as the carrier gas. Quality control samples were produced by pooling different samples and analysed along with the other samples. PCA plot showed a clear clustering of QC samples at the center of all experimental samples, indicating reproducible sample analysis (Fig. S5). Metabolites were identified and quantified using a workflow described by Behrends et al. (39). While the initial sample production and data acquisition were initially performed on five independent replicates, the first one did not include any contamination checks, while the last four ones did. Hence, we decided to include only the four replicates based on a contamination-free quality check. A sample of whole larvae at 12 h was also excluded from the analysis because of failed derivatization.

### Cholesterol supplementation

Cholesterol solutions were prepared by dissolving 100 mg of cholesterol (C75209; Sigma) in 6 mL of acetone. The solution was sterilized using a 0.2 µm filter, evaporated overnight inside a biosafety cabinet, and subsequently ground into a fine powder. This powder was resuspended in 25 mL of autoclaved food suspension to obtain a 4 mg/mL cholesterol stock solution. A control solution was prepared following the same procedure, omitting cholesterol. The stock solution was then diluted 1:3, 1:30, or 1:300, and 40 µL of each dilution was administered to larvae, resulting in final concentrations of 106, 35.6, 3.6, or 0.36 µg/mL, respectively.

### Gnotobiotic larvae preparation and follow-up

Axenic larvae were generated as described above. First-instar larvae were individually transferred to 24-well plates. Overnight bacterial cultures (auxotrophic HA416, wild-type MG1655, ∆*fadE* FBE640 or ∆*fadH* FBE765 *E. coli* strains) were centrifuged and resuspended to a final concentration of ~10⁸ CFU/mL for all strains or ~10³ CFU/mL for MG1655, FBE640, and FBE765, before being added to the larvae. Larvae mono-associated with the auxotrophic HA416 strain were either supplemented with 12.5 μg/mL m-DAP and 50 μg/mL D-Ala or left unsupplemented. Plates were maintained in a climatic chamber at 80% relative humidity under a 12:12 h light/dark cycle (30°C/25°C). Larval development was assessed daily, and the developmental stage of each individual was recorded over a 14-day period.

### Statistics and data analysis

Data were analysed using MetaboAnalyst (version 6.0) (40). All statistical results can be found in Table S2. Prior to normalization, geometric means of all metabolite peak intensities were calculated for each sample, and conditions were compared with a two-sided paired *t*-test. Then, a total peak intensity normalization was performed for each sample to account for differences in the amount of material input. Data were normalized by square root transformation and auto scaling. Quality log2-transformed ratios between germ-free and colonized conditions were calculated for each type of sample at each time point and each replicate, and analysed by *t*-test for analyses and Volcano plots. Paired ratios were calculated, and a log_2_ transformation was applied. For volcano plots and KEGG pathway enrichment analysis, thresholds were set at *P* = 0.1 maximum and |log_2_(FC)| = 0.3 minimum (30% enriched). Metabolomic data are inherently variable, and biological replicates are typically prepared by pooling individuals collected within the same experiment. Our experimental design comprised five independent experiments, each replicate consisting of pools of 50–60 larvae or dissected guts. This experimental design can increase between-replicate variability, resulting in a relatively high noise level across replicates. However, this high number of independent replicates increases confidence in statistically significant findings: in a high-noise data set, a result that nonetheless reaches statistical significance is less likely to represent a false positive or experimental artifact, and more likely to reflect a genuine biological signal. Hence, no correction was applied to *t*-tests. This statistical analysis (as any) thus needs to be considered with caution, but this strategy allowed us to keep a sufficient number of metabolites of interest to look at pathway enrichment. As many metabolites are part of several pathways, we consider that a pathway’s enrichment can be determined if it contains at least four metabolites detected in the data set (Table S1). Metabolomic pathways were scrutinized using the Kyoto Encyclopedia of Genes and Genomes (KEGG). Analysis of developmental success, CFU counts, and larval development duration were performed with generalised linear mixed models (GLMM) or a linear mixed-effect model (lmer) using the lme4, lmerTest, and lsmeans packages in R (version 4.3.0). For developmental success data, an ANOVA was performed on a logistic regression (glmer), while for CFU counts, an ANOVA was performed on a linear regression (lmer). The replicate was set as a random effect in both cases.

## SUPPLEMENTAL MATERIAL

Figure S1 mbio.01035-26-s0001.tif PCA plots on metabolomic analysis.

Figure S2 mbio.01035-26-s0002.tif Volcano plots on metabolomic analysis.

Figure S3 mbio.01035-26-s0003.tif Larval development with non-growing bacteria.

Figure S4 mbio.01035-26-s0004.tif Colonization with bacteria deficient for fatty acid β-oxidation.

Figure S5 mbio.01035-26-s0005.tif Principal component analysis of the metabolomic analysis including quality controls.

Supplemental legends mbio.01035-26-s0006.docx Supplemental figure and table legends.

Table S1 mbio.01035-26-s0007.xlsx Metabolites and their raw peak intensities.

Table S2 mbio.01035-26-s0008.xlsx Statistical analysis results.

ASM does not own the copyrights to Supplemental Material that may be linked to, or accessed through, an article. The authors have granted ASM a non-exclusive, world-wide license to publish the Supplemental Material files. Please contact the corresponding author directly for reuse.
