# Supplementary figures and images for: The microbiota affects energy production, nitrogen excretion, and sterol metabolism in mosquito larvae
Source: mBio. 2026 Jun 12;17(7):e01035-26. doi: 10.1128/mbio.01035-26 (PMC13343993; doi:10.1128/mbio.01035-26)

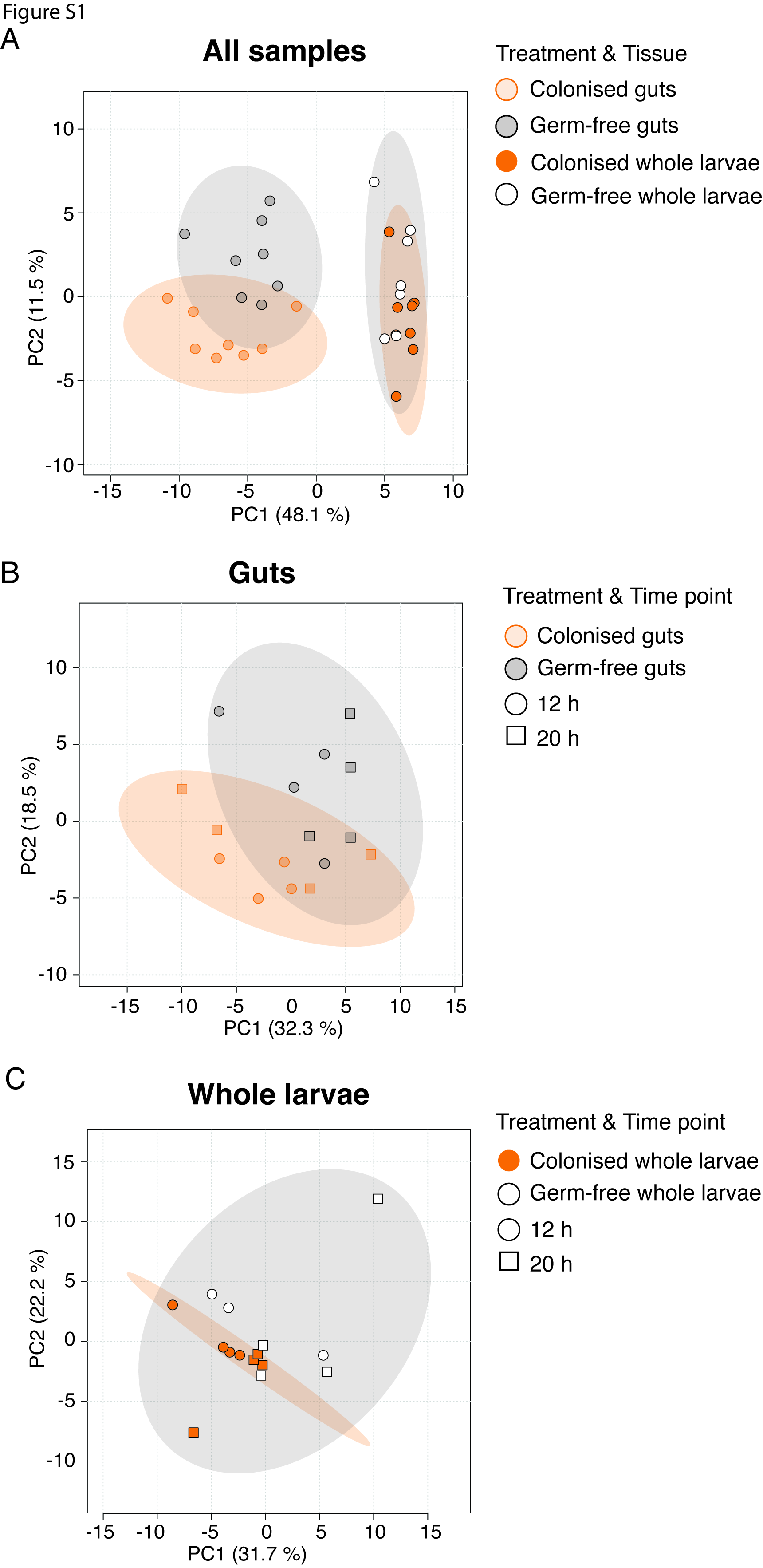

Supplement: Figure S1 — PCA plots on metabolomic analysis. [file mbio.01035-26-s0001.tif]

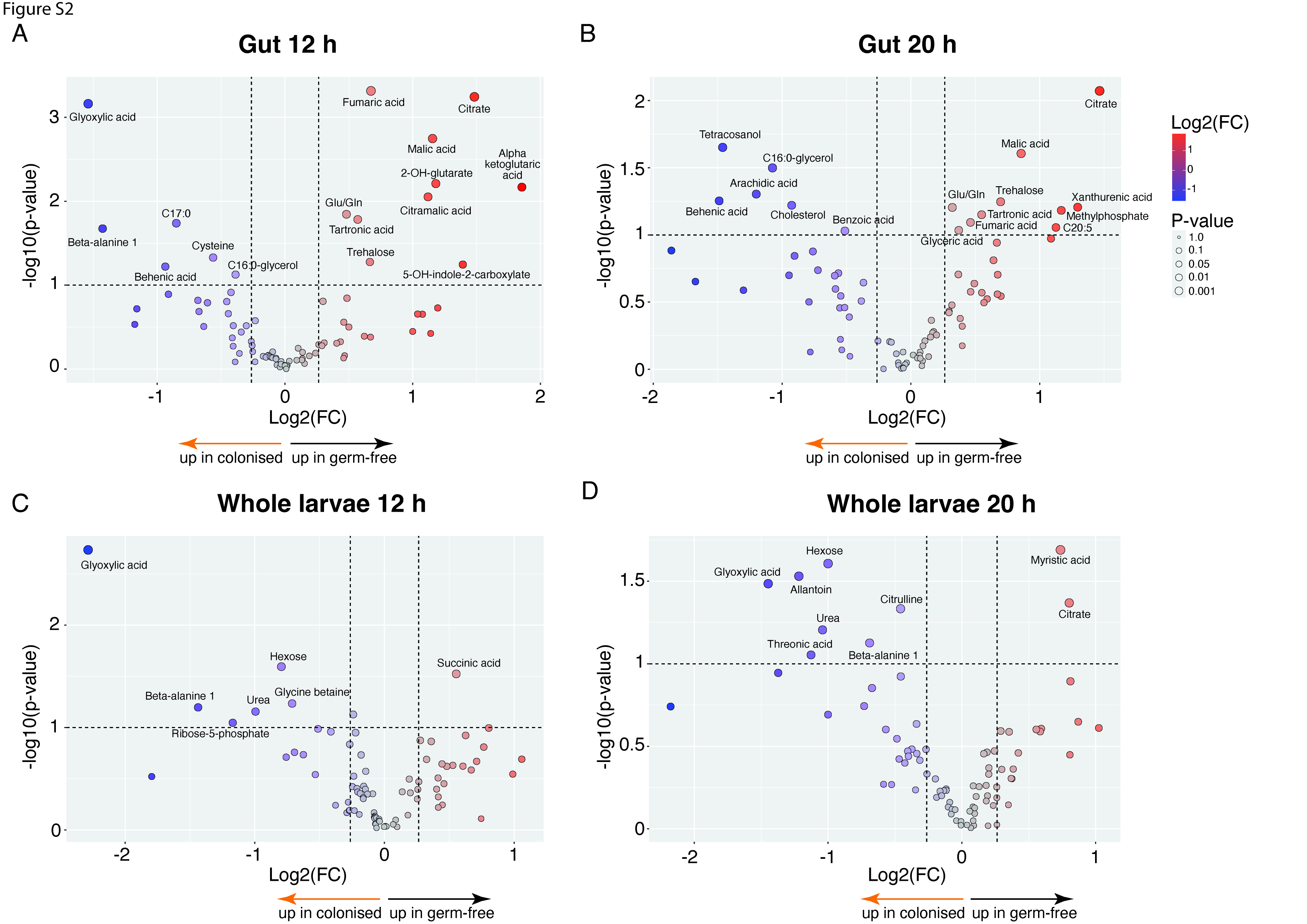

Supplement: Figure S2 — Volcano plots on metabolomic analysis. [file mbio.01035-26-s0002.tif]

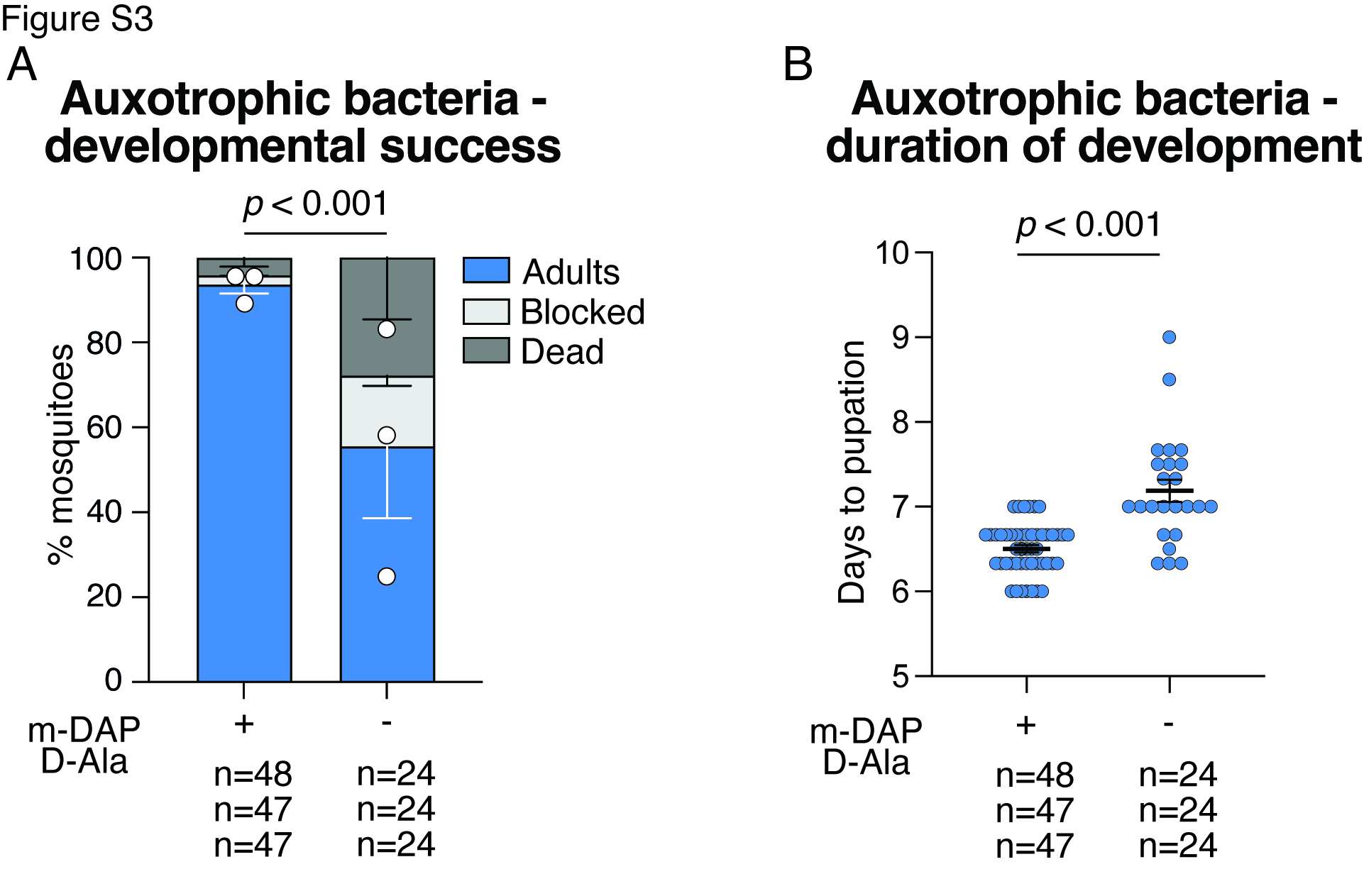

Supplement: Figure S3 — Larval development with non-growing bacteria. [file mbio.01035-26-s0003.tif]

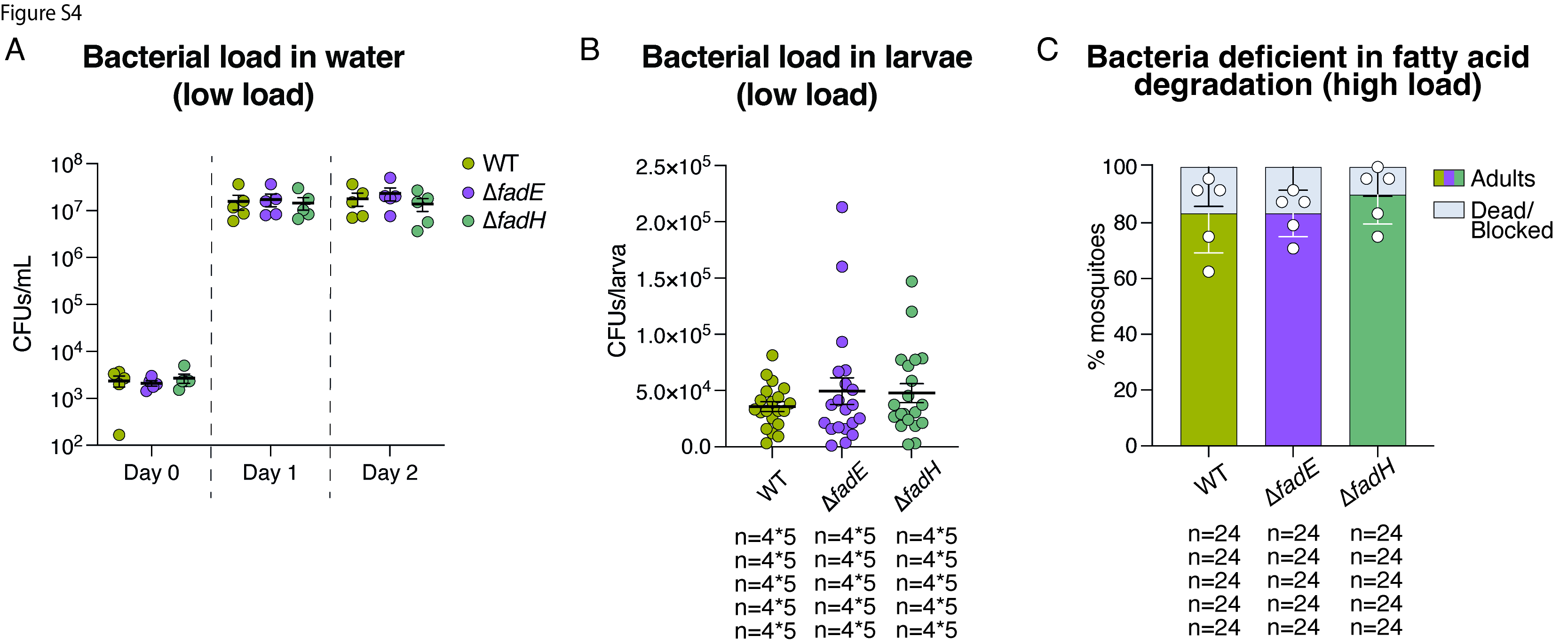

Supplement: Figure S4 — Colonization with bacteria deficient for fatty acid β-oxidation. [file mbio.01035-26-s0004.tif]

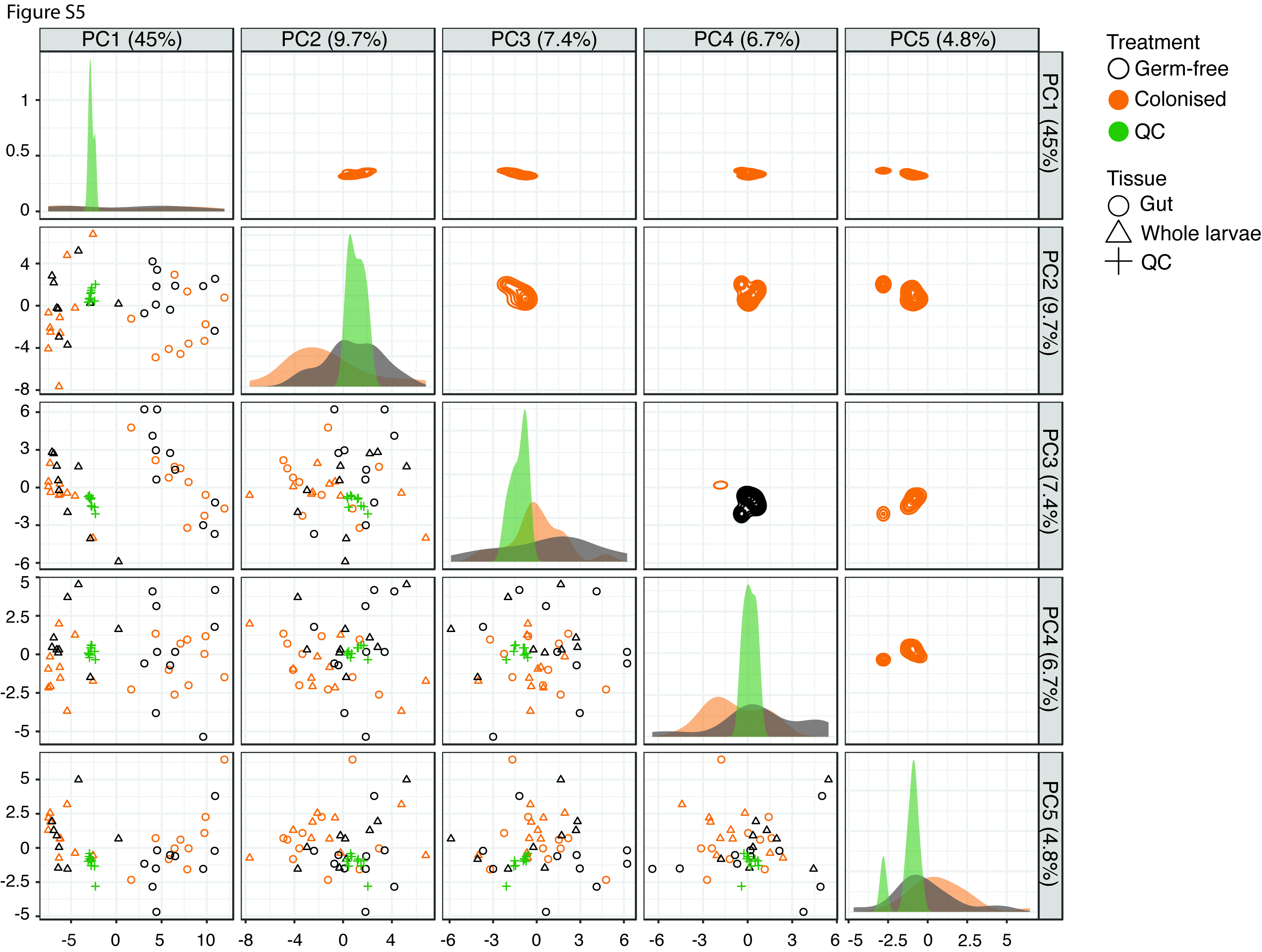

Supplement: Figure S5 — Principal component analysis of the metabolomic analysis including quality controls. [file mbio.01035-26-s0005.tif]
