# Supplementary material for: The microbiota affects energy production, nitrogen excretion, and sterol metabolism in mosquito larvae
Source: mBio. 2026 Jun 12;17(7):e01035-26. doi: 10.1128/mbio.01035-26 (PMC13343993; doi:10.1128/mbio.01035-26)
Supplement: Supplemental legends — Supplemental figure and table legends. [file mbio.01035-26-s0006.docx]

**Figure S1. Principal Component Analysis (PCA) of gut and larval metabolomes.** (**A**) PCA plot showing a clear separation along PC1 between gut (transparent symbols) and whole larva (solid symbols) metabolomes (PERMANOVA tissue: F = 22.7, *p* = 0.001). Samples are also partially grouped by colonization status (orange: colonized, white/grey: germ-free; PERMANOVA colonization: F = 2.0, *p* = 0.085). (**B**) PCA of gut metabolomes, with partial separation by colonization but not by time-point (circles: 12 h; squares: 20 h; PERMANOVA colonization: F = 2.6, *p* = 0.01; time-point: F = 1.1, *p* = 0.39). (**C**) PCA of whole larva metabolomes, showing partial separation between colonized and germ-free samples and between 12 h and 20 h samples (PERMANOVA colonization: F = 1.9, *p* = 0.039; time: F = 2.1, *p* = 0.023). In each panel, grey and orange ellipses display 95 % confidence regions of germ-free and colonised samples, respectively.

**Figure S2. Metabolites significantly affected by bacterial colonization in gut and larval samples at different time points.** Volcano plots showing differential metabolite abundances between colonized and germ-free conditions in gut (**A**-**B**) and whole-larva (**C**-**D**) samples collected at 12 h (**A**,**C**) or 20 h (**B**,**D**) post bacterial decolonization. Metabolites enriched in colonized samples are indicated by orange arrows; those enriched in germ-free samples by black arrows. A fold change threshold of 1.2 and a t-test *p* value threshold of 0.1 were applied. Both fold changes and *p* values are log-transformed for visualization.

**Figure S3. Role of bacteria proliferation on mosquito larval development**. (**A**) Development of larvae colonised with the auxotrophic *E. coli* HA416 strain as a function of m-DAP and D-Ala supplementation. Bar plots show the proportions of adults (blue), developmentally blocked (light grey) or dead larvae/pupae (dark grey). Statistical significance on the proportion of adults was assessed using a generalized linear mixed model using the proportion of adults and the m-DAP and D-Ala supplementation as fixed factors, and the replicate as a random factor. (**B**) Developmental duration of larvae colonized with the auxotrophic *E. coli* HA416 strain as a function of m-DAP and D-Ala supplementation. Plots show results from three independent replicates. The number of individuals analysed in each replicate is indicated below the plots. Statistical significance was determined using a linear mixed-effect model using the duration of larval development and m-DAP and D-Ala supplementation as fixed factors, and the replicate as a random factor.

**Figure S4.** ***fadE* and *fadH* mutants are not deficient in colonizing breeding water and larvae and not pathogenic to larvae.** (**A**) CFUs of wild type (WT, khaki), ∆f*adE* (purple) or ∆*fadH* (green) *E. coli* in larval breeding water at the time of colonization, 1 day and 2 days later. (**B**) CFU quantification of WT (khaki), ∆f*adE* (purple) or ∆*fadH* (green) *E. coli* in fourth-instar larvae. Statistical significance on the CFUs was assessed using a linear model with bacterial strain and CFUs as fixed factors and the replicate as a random factor. (**C**) Development of larvae when providing a high quantity of bacteria at the start of the experiment (10^8^ CFUs/mL). Bar plots show the proportions of adults (khaki, purple or green) and developmentally blocked or dead larvae (grey). Statistical significance on the proportion of adults was assessed using a generalized linear mixed model using the proportion of adults and the bacterial strain as fixed factors, and the replicate as a random factor. All plots show results from five independent replicates. The number of individuals analysed in each replicate is indicated below the plots.

**Figure S5.** **Principal Component Analysis (PCA) of gut and larval metabolomes and quality control (QC) samples.** PCA plot showing the distribution of experimental and QC sample along the first five principal components. Sample type is colour-coded: colonized (orange), germ-free (white), and QC (green). Time points are indicated by symbol shape: circles for 12 h, squares for 20 h, and crosses for QC samples.

**Supplementary table legends**

**Table S1. List of metabolites and their raw peak intensities.** For each metabolite, its general category, retention time (RT) and intensity in each sample is indicated. Values in grey belongs to the first replicate that was excluded because lacking appropriate contamination checks. Values in red belong to the sample that did not derivatize. QC: quality control.

**Table S2. Statistical analysis results.** For Figures 4 and S2, the fold change values (and their the log_2_ transformations) and the raw t-test *p* values (and their -log_10_ transformations) are shown for the metabolites with *p* < 0.1 and -1.2 < fold change < 1.2. Metabolites significantly affected by bacterial decolonization in both 12 h and 20 h time-points are highlighted in green.

For Figure 7B-C, GLMM results are shown for models using the proportion of blocked larvae, dead larvae, dead pupae and adults, and the cholesterol concentration as fixed factors, and the replicate as a random factor.

For Figure 7D, GLMM results are shown for the model using the proportion of adults and the bacterial strain as fixed factors, and the replicate as a random factor.

For Figure S1, PERMANOVA F, R-squared and *p* value results are indicated for each discriminatory variable (tissue, time, colonization, time, colonization*time). Significant *p* values are highlighted in red.

For Figure S3, GLMM and LMM results are showed for models using the proportion of blocked larvae, dead larvae, dead pupae and adults (Figure S3A) or the duration of larval development (Figure S3B) and the m-DAP/D-Ala supplementation as fixed factors, and the replicate as a random factor. Significant *p* values are highlighted in red.

For Figure S4, LMM and GLMM results are showed for models using the number of CFUs in water (Figure S4A) or larvae (Figure S4B) or the proportion of adults (Figure S4C) and the bacterial strain as fixed factors, and the replicate as a random factor. Significant *p* values are highlighted in red.
